# MicroRNA-20b promotes cell growth of breast cancer cells partly via targeting phosphatase and tensin homologue (PTEN)

**DOI:** 10.1186/2045-3701-4-62

**Published:** 2014-10-14

**Authors:** Weidong Zhou, Guixiu Shi, Qiuyan Zhang, Qiuwan Wu, Boan Li, Zhiming Zhang

**Affiliations:** Center Laboratory, The First Affiliated Hospital of Xiamen University, Xiamen, Fujian 361003 PR China; School of Life Sciences, Xiamen University, Xiamen, Fujian 360112 PR China; Department of Rheumatology and Clinical Immunology, The First Affiliated Hospital of Xiamen University, Xiamen, Fujian 361003 PR China; Department of Breast Surgery, The First Affiliated Hospital of Xiamen University, Xiamen, Fujian 361003 PR China

**Keywords:** Breast cancer, MiR-20b, PTEN, Posttranscriptional regulation

## Abstract

**Background:**

MicroRNAs (miRNAs) are endogenous, small non-coding RNAs that play important roles in multiple biological processes. MiR-20b has been reported to participate in breast cancer tumorigenic progression, however, the functional roles are still unclear and under debating. The aim of this study is to explicit the molecular mechanism of miR-20b underlying breast cancer tumorigenesis.

**Results:**

In the present study, we showed that miR-20b was overexpressed in human breast cancer tissues and cell lines compared with paired adjacent normal tissues and normal cell lines, respectively. We identified PTEN, a well-known tumor suppressor, as the functional downstream target of miR-20b. Luciferase assays confirmed that miR-20b could directly bind to the 3′ untranslated region(UTR) of PTEN and suppress translation. Alteration of miR-20b expression changed PTEN protein level but not mRNA expression in ZR-75-30 and MCF-7 breast cancer cells, suggesting miR-20b regulates PTEN gene expression at the posttranscriptional level. Furthermore, upregulation of miR-20b significantly promoted the proliferation, colony formation and DNA synthesis of ZR-75-30 and MCF-7 breast cancer cells. Conversely, knockdown of miR-20b expression inhibited the growth of breast cancer cells *in vitro* and *in vivo*.

**Conclusion:**

Dysregulation of miR-20b plays critical roles in the breast cancer tumorigenesis, at least in part via targeting the tumor suppressor PTEN. This microRNA may serve as a potential diagnostic marker and therapeutic target for breast cancer.

**Electronic supplementary material:**

The online version of this article (doi:10.1186/2045-3701-4-62) contains supplementary material, which is available to authorized users.

## Background

Breast cancer is the most frequently diagnosed malignancy and the leading cause of cancer death in women worldwide. Currently, the large number of etiological factors and the complexity of breast cancer present challenge for prevention and treatment. Breast cancer tumorigenesis can be described as a multi-step process in which a normal cell undergoes malignant transformation to a fully developed tumor through accumulations of genetic and epigenetic changes [1]. Although alterations in many oncogenic and tumor-suppressive genes are reportedly implicated in breast cancer, the molecular mechanisms that maintain the malignant proliferation of tumor cells remains poorly understood.

MicroRNAs (miRNAs) are endogenous, small non-coding RNAs that regulate gene expression at the posttranscriptional level [2]. These miRNAs pair with partially complementary sites in the 3′-untranslated regions (UTRs) of target mRNAs, leading to translational repression and/or mRNA degradation. MiRNAs participate in a wide array of biological processes including development and differentiation, cell proliferation, apoptosis, and metabolism [3, 4]. Aberrant miRNA expression has been frequently observed in various human tumors, indicating an important role of miRNAs in tumorigenesis [5].

Recently, studies of miRNA profiles found several deregulated miRNAs in breast cancer, including miR-20b [6, 7]. MiR-20b belongs to the miR-106a-363 cluster, together with miR-17-92 and miR-106b-25 clusters, form a large family of highly similar miRNAs called the miR-17 family [8]. In many human malignancies, members of the miR-17 family have been reported to accumulate in tumor cells and speculated to have an oncogenic role [9, 10]. The probability of survival was significantly lower in gastric cancer patients with high expression levels of miR-20b [11]. MiR-20b was also reported to favor the survival of tumor cells upon the oxygen supply [12]. These studies suggested that miR-20b can serve as a potential oncogene. MiR-20b was also reported to involve regulation of VEGF expression by targeting HIF-1α and STAT3 in MCF-7 breast cancer cells [13]. A newly-published study has shown that ionizing radiation, a potent tumor-causing agent, can induce miR-20b expression in rat mammary gland tissues, and the transcription factor early growth response-1 involves in the regulation of miR-20b in the breast cancer cell line HCC1806 [14].

The present study also showed that miR-20b was upregulated in human breast cancer tissues, and a bioinformatics analysis predicted phosphatase and tensin homologue (PTEN) to be a potential target of miR-20b. PTEN gene, a well-known anti-oncogene, encodes a phosphatase that converts phosphatidylinositol 3,4,5-trisphosphate to phosphatidylinositol 4,5-bisphosphate, thereby antagonizing the highly oncogenic PI3K/Akt-signaling pathway [15]. PTEN expression is frequently altered in a variety of human cancers [16], and subtle changes in PTEN dose dictate critical outcomes in tumor initiation and progression *in vivo* [17]. Recent evidence showed that regulation of the PTEN-PI3K-Akt pathway by miRNAs plays a crucial role in breast cancer progression [18], which indicating a novel way to investigate the tumorigenesis, diagnosis, and therapy of breast cancer. In this study, we aim to explicit the molecular mechanism of miR-20b underlying breast cancer tumorigenesis.

## Results

### Inverse level of miR-20b and PTEN in breast cancer

**Figure 1:**
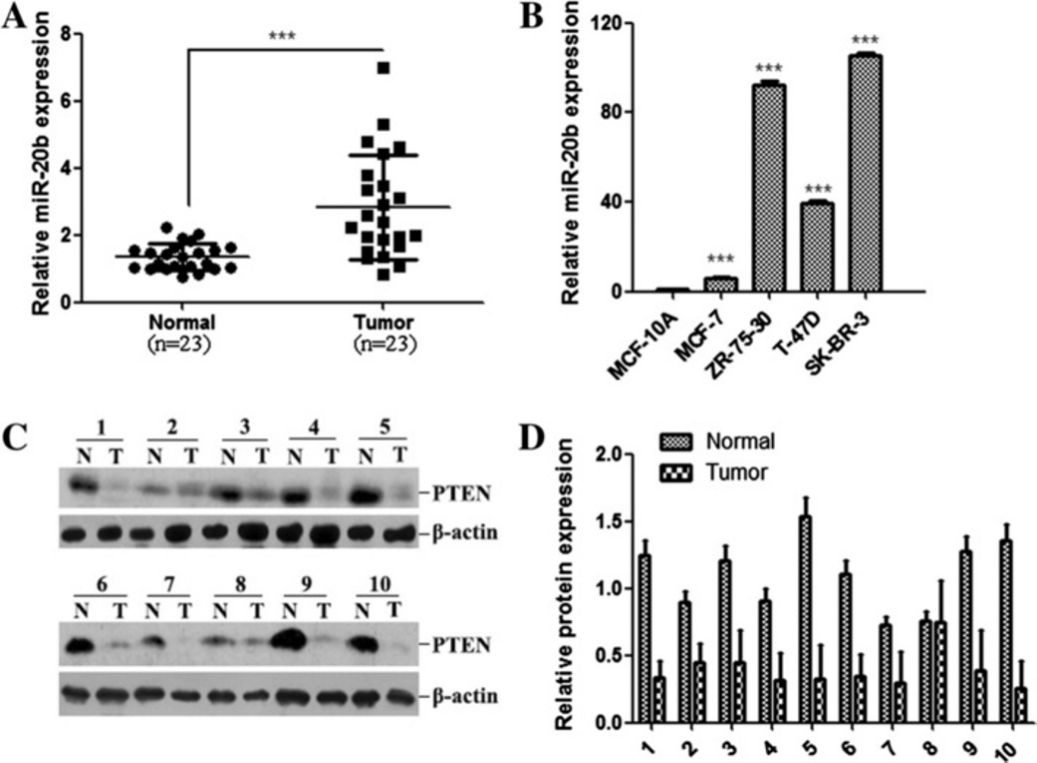
**Expression of miR-20b is associated with PTEN level. (A)** qRT-PCR analysis of miR-20b expression in 23 paired breast tumor tissues (tumor) and their adjacent normal tissues (normal). ***, *P* < 0.001. **(B)** The expression of miR-20b was examined in nontumorigenic breast epithelial cells (MCF-10A) and breast cancer cell lines, including MCF-7, ZR-75-30, T-47D and SK-BR-3. The miR-20b expression was normalized by U6 expression. ***, *P* < 0.001 compared with MCF-10A. **(C)** Western blot analysis of PTEN protein expression between the breast tumor and normal tissues. N, normal breast tissue; T, breast tumor tissues. **(D)** Quantitative analysis of PTEN protein expression. PTEN expression was normalized by β-actin.

We analyzed the expression level of miR-20b in 23 paired clinical breast cancer tissues and the adjacent normal breast tissues by miRNA qRT-PCR. The average expression level of miR-20b was significantly increased in breast cancer tissues compared with paired normal adjacent tissues (Figure 1A; *P* < 0.001). We further assessed the expression level of miR-20b in a panel of breast cancer cell lines. Compared with the normal mammary epithelial cell line (MCF-10A), miR-20b expression level was upregulated in all four examined breast cancer cell lines (MCF-7, ZR-75-30, T-47D and SK-BR-3) (Figure 1B). In addition, among the above 23 paired clinical breast cancer tissues and the adjacent normal breast tissues, we randomly selected 10 patients for further detecting the PTEN protein expression by western blot. The results showed that the PTEN protein level was downregulated in human breast cancer tissues compared with in the matching normal breast tissues (Figure 1C and D). These data indicate that the protein level of PTEN is negatively correlated with the miR-20b expression (Additional file 1).

### MiR-20b binds directly to the 3′-UTR of PTEN

**Figure 2:**
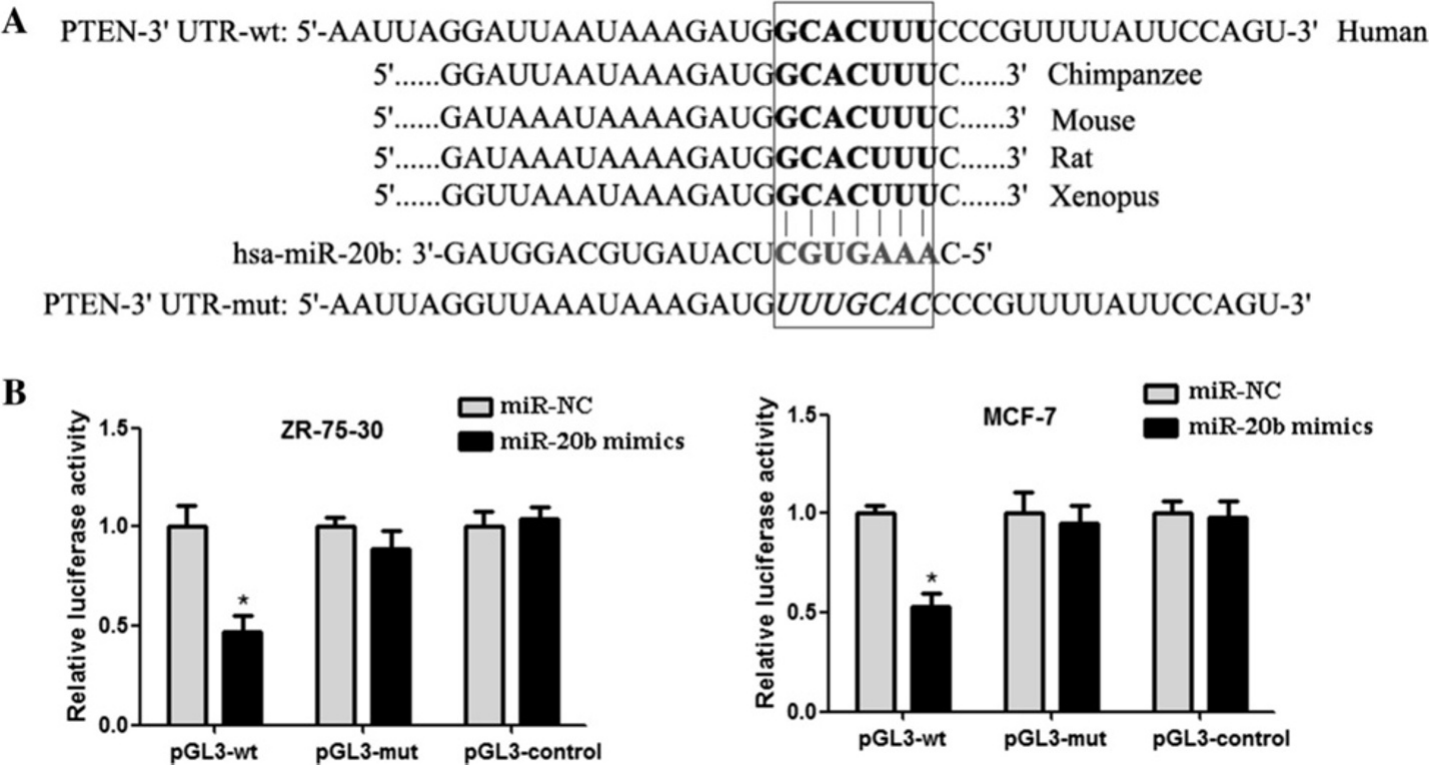
**MiR-20b directly targets the 3′-UTR of PTEN. (A)** Sequence alignment of the miR-20b base-paring site in the 3′-UTR of PTEN mRNA. The region complementary to the seven nucleotides of miR-20b is highly conserved among human, chimpanzee, mouse, rat, and xenopus. The “seed” sequence of miR-20b that is complementary to PTEN is shown in the box. The mutant sequence (PTEN-3′-UTR-mut) is identical to the wild-type sequence (PTEN-3′-UTR-wt) except the mutated nucleotides are shown in italics. **(B)** Luciferase assays in ZR-75-30 and MCF-7 breast cancer cells. Cells were cotransfected with wt/mut 3′-UTR with miR-20b mimics or negative control as indicated. Forty-eight hours after transfection, luciferase activity was detected using Dual-Luciferase Reporter Assay System according to the manufacturer’s instruction. Error bars represent mean ± SD from three independent experiments. *, *P* < 0.05 when compared with corresponding negative control.

To explore the molecular mechanism of miR-20b in the growth of breast cancer cells, we used a bioinformatics analysis to search for putative protein-coding gene targets of miR-20b, especially for those that can affect cancer cell growth. According to this rationale, PTEN was selected as the candidate target gene of miR-20b, which was highly conserved among different species and whose 3′-UTR of mRNA contained a complementary site for the seed region of miR-20b (Figure 2A). Furthermore, we performed a luciferase reporter assay to verify that miR-20b directly targets PTEN. We subcloned PTEN 3′-UTR sequences containing the predicted target site (wild type, pGL3-wt) of miR-20b or mutated sequences (mutant type, pGL3-mut) into the pGL3-Control vector, respectively. The results showed that co-transfection of miR-20b mimics and pGL3-wt significantly decreased the luciferase activities in breast cancer cells (ZR-75-30 and MCF-7) but not that of the mutant reporter (Figure 2B), indicating that miR-20b binds directly to the 3′-UTR of PTEN.

### MiR-20b downregulates PTEN expression at the posttranscriptional level

**Figure 3:**
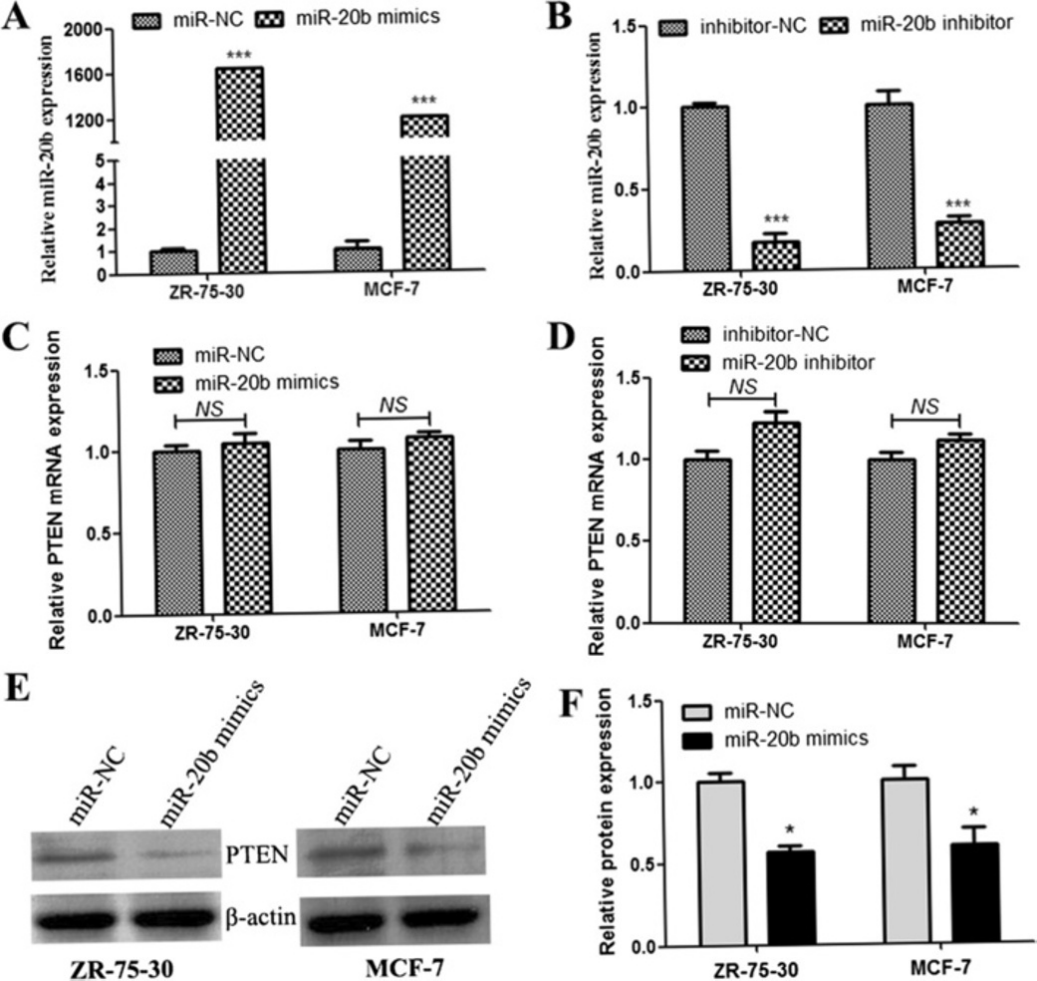
**MiR-20b inhibits PTEN protein expression. (A-B)** MiR-20b expression was detected by qRT-PCR. ZR-75-30 and MCF-7 breast cancer cells transfected with miR-20b mimics showed a significant increase in miR-20b expression **(A)**, while the cells transfected with miR-20b inhibitor resulted in a significant decrease of miR-20b expression **(B)**. **(C-D)** qRT-PCR analysis of PTEN mRNA expression. ZR-75-30 and MCF-7 breast cancer cells transfected with miR-20b mimics or inhibitor did not affect PTEN mRNA expression. **(E)** Representative pictures of PTEN protein expression detected by western blot. **(F)** Quantitative analysis showed that PTEN protein expression in breast cancer cells transfected with miR-20b mimics decreased when compared with the negative control. Error bars represent mean ± SD from three independent experiments. *, *P* < 0.05; ***, *P* < 0.001. *NS*, no significant differences.

To evaluate the effect of miR-20b on PTEN expression level, we transfected the breast cancer cell lines ZR-75-30 and MCF-7 with miR-20b mimics or inhibitor. Compared with miR-20b mimic negative control (miR-NC), transfection with 50 nM of miR-20b mimics in ZR-75-30 and MCF-7 cells led to more than 1000-fold increase in miR-20b expression as detected by quantitative real-time PCR (Figure 3A). In contrast, ZR-75-30 and MCF-7 cells transfected with 100 nM of miR-20b inhibitor dramatically decreased miR-20b expression level as compared with that of negative control (inhibitor-NC) transfected cells (Figure 3B). After transfecting with miR-20b mimics or inhibitor, no significant difference in PTEN mRNA level was observed in the both breast cancer cells (Figure 3C and D). However, miR-20b could significantly downregulate PTEN protein level in ZR-75-30 and MCF-7 breast cancer cells (Figure 3E and F). These data suggest that miR-20b can downregulate PTEN gene expression at the posttranscriptional level.

### MiR-20b enhances breast cancer cell proliferation

**Figure 4:**
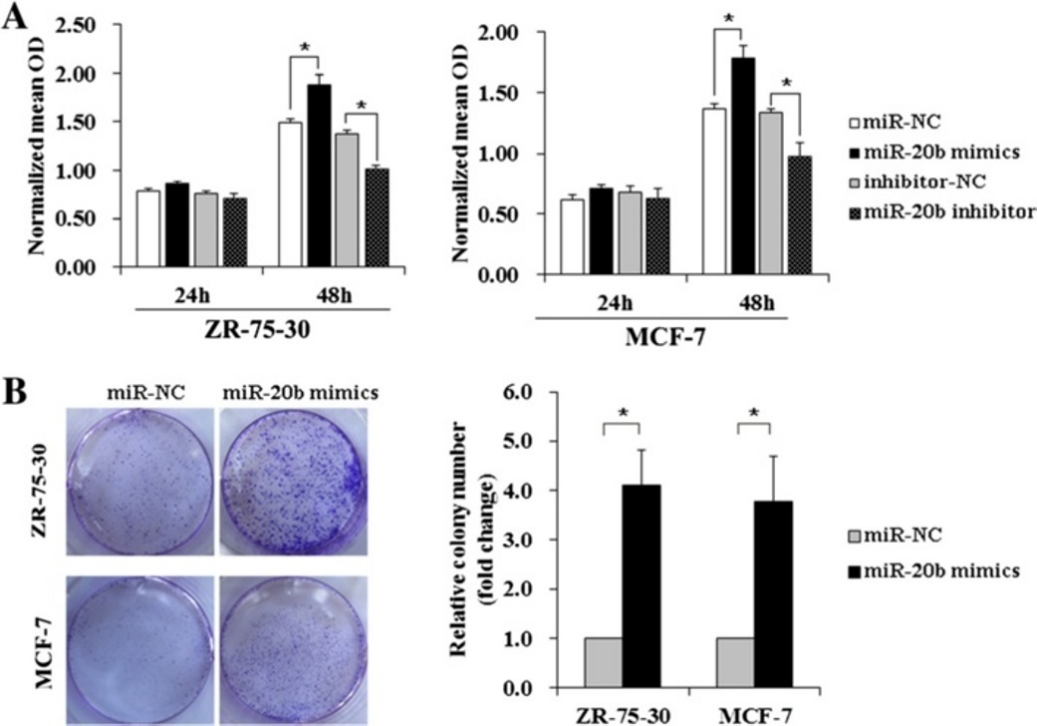
**Effects of miR-20b on proliferation of breast cancer cells. (A)** ZR-75-30 (left-panel) and MCF-7 (right-panel) breast cancer cells were transfected with miR-20b mimics and miR-20b inhibitor. MTS assays were used to detect the cell viability after the indicated periods. **(B)** The colony formation assays showed that upregulation of miR-20b in ZR-75-30 and MCF-7 breast cancer cells significantly enhanced their growth ability, as indicated by the increase in colony numbers. Each bar represents the mean of three independent experiments. *, *P* < 0.05.

To investigate the effects of miR-20b on breast cancer tumorigenesis, we first assessed the effects of its expression on cell viability by using the MTS assay. We transfected miR-20b mimics into breast cancer cells ZR-75-30 and MCF-7, which showed that overexpression of miR-20b significantly increased the cell viabilities of both breast cancer cells at 48 h after transfection in comparison with that of negative control (miR-NC) transfected cells (*P* < 0.05) (Figure 4A). Whereas miR-20b inhibition dramatically restrained the cell viabilities of ZR-75-30 and MCF-7 at 48 h after transfection as compared with that of negative control (inhibitor-NC) transfected cells (*P* < 0.05) (Figure 4A). Furthermore, upregulation of miR-20b in ZR-75-30 and MCF-7 breast cancer cells significantly enhanced their growth ability, as indicated by the increase in colony numbers (*P* < 0.05) (Figure 4B). In addition, to analyze the cell cycle distribution, DNA content was determined by flow cytometry using PI staining. We found that the G_0_/G_1_ phase was increased in miR-20b inhibitor-transfected MCF-7 breast cells compared with control cells. The proportion of cells corresponding to the proportion of cells in the S and G_2_/M phase was lower in miR-20b inhibitor-transfected MCF-7 cells than that in control cells. It seems that knockdown of miR-20b expression resulted in cells that were blocked in G_0_/G_1_ phase and could not enter into S phase for DNA synthesis. Further analysis revealed that knockdown of miR-20b significantly decreased the proliferation index of MCF-7 cells (Additional file 2).

### MiR-20b promotes DNA synthesis of breast cancer cells

**Figure 5:**
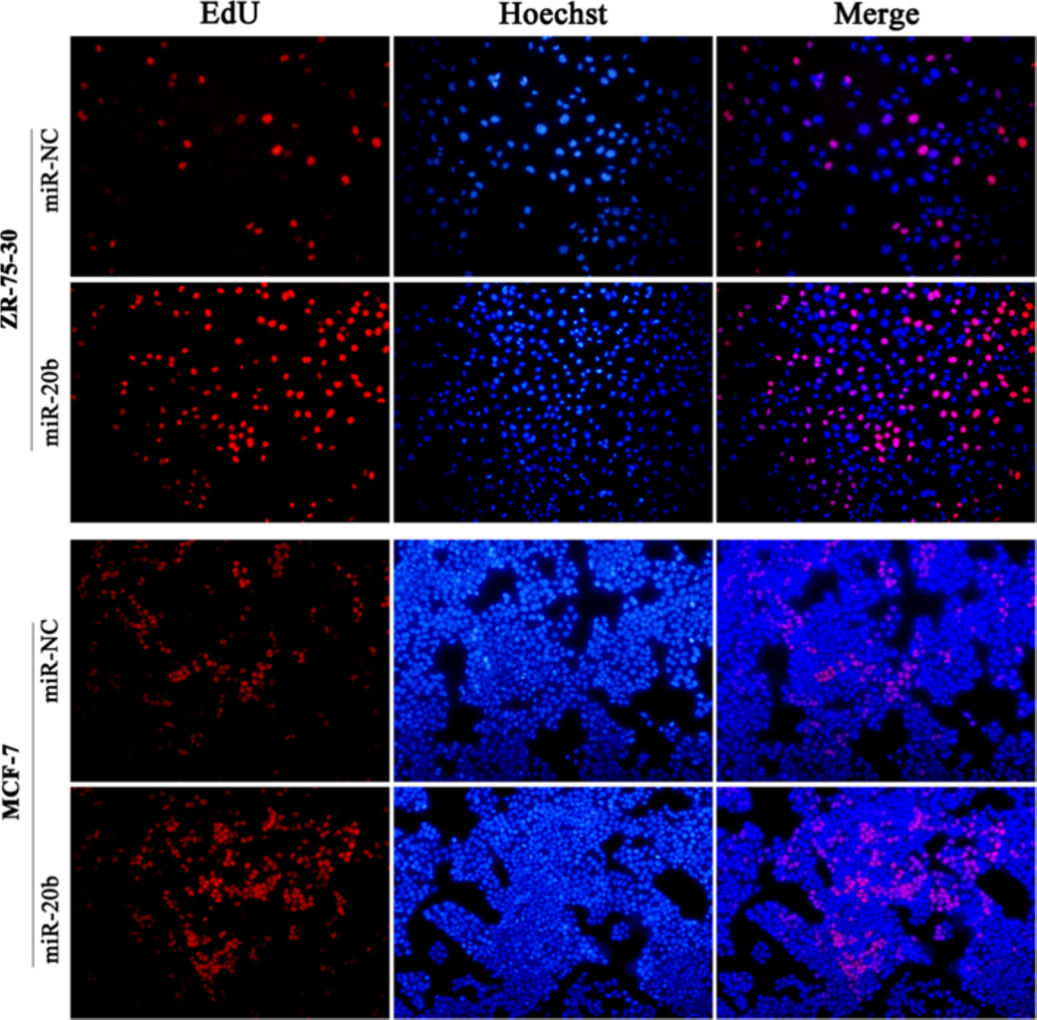
**Upregulation of miR-20b promotes breast cancer cell proliferation through enhancing DNA synthesis.** ZR-75-30 cells (top-panel) and MCF-7 cells (below-panel) were transfected with miR-20b mimics or negative control; 48 hours after transfection, EdU labeling assays were performed. Representative micrographs (200×) and quantification of EdU incorporating-cells after transfection with miR-20b mimics or negative control.

**Figure 6:**
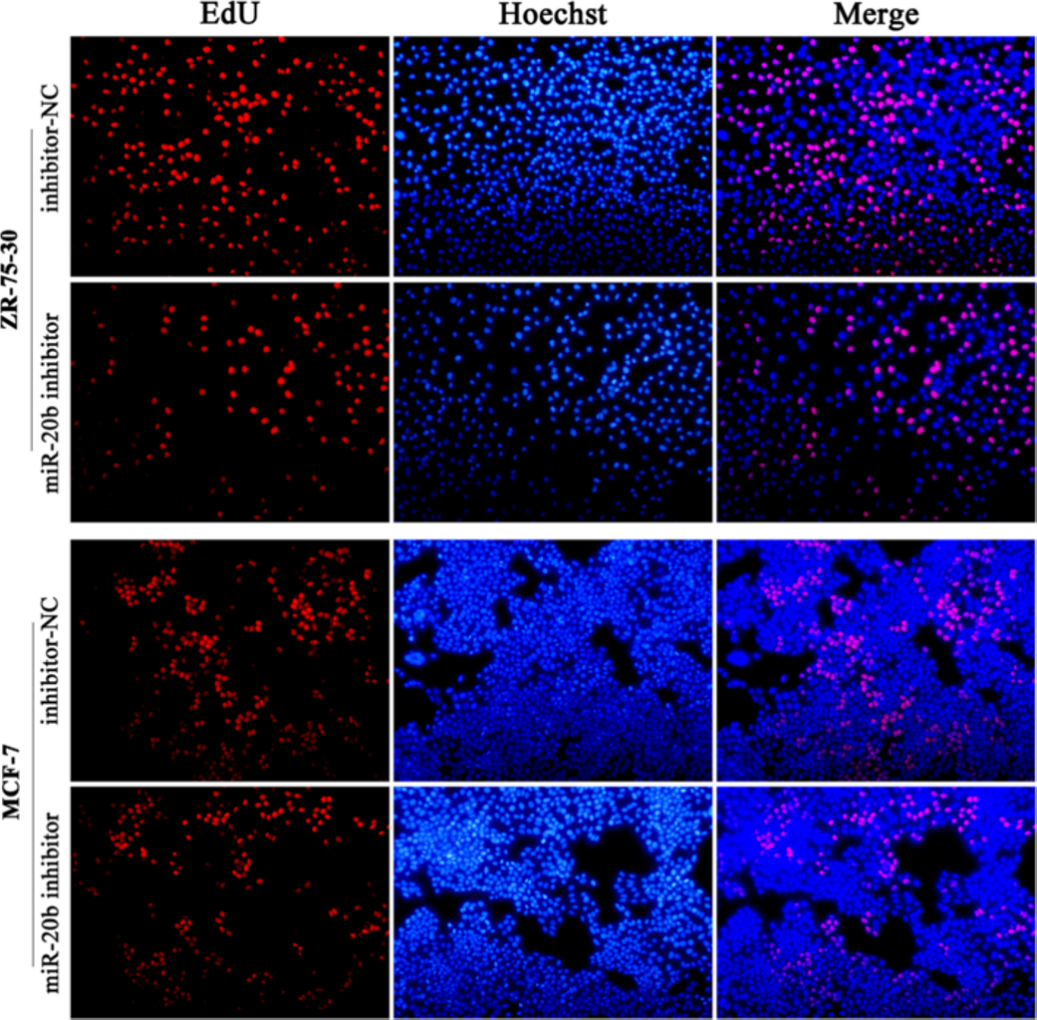
**Inhibition of miR-20b suppresses the proliferation of breast cancer cells.** ZR-75-30 cells (top-panel) and MCF-7 cells (below-panel) were transfected with miR-20b inhibitor or negative control; 48 hours after transfection, EdU labeling assays were performed. Representative micrographs (200×) and quantification of EdU incorporating-cells after transfection with miR-20b inhibitor or negative control.

To further investigate the ability of miR-20b to promote cell proliferation, the 5-ethynyl-2′-deoxyuridine (EdU) incorporation assays were performed. The results showed that miR-20b overexpression enhanced DNA synthesis in both ZR-75-30 and MCF-7 breast cancer cells. The percentage of miR-20b mimics transfected ZR-75-30 cells (33.62% ± 2.14%) and of miR-20b mimics transfected MCF-7 cells (29.08% ± 3.61%) showed newly synthesized DNA by the EdU incorporation assays were significantly higher than that of ZR-75-30 control cells (25.61% ± 3.61%) and MCF-7 control cells (19.82% ± 4.41%) (Figure 5; *P* < 0.05). Meanwhile, miR-20b inhibitor suppressed DNA synthesis in both ZR-75-30 and MCF-7 breast cancer cells. The percentage of EdU positive cells in miR-20b inhibitor transfected ZR-75-30 cells (35.51% ± 2.24%) and MCF-7 cells (25.79% ± 3.95%) were significantly decreased as compared with that of in negative control transfected ZR-75-30 cells (40.47% ± 4.73%) and MCF-7cells (30.88% ± 3.52%) (Figure 6; *P* < 0.05). Collectively, these results support the role of miR-20b in the promotion of breast cancer cell proliferation, suggesting that miR-20b has an oncogene function.

### MiR-20b knockdown suppresses tumor growth *in vivo*

**Figure 7:**
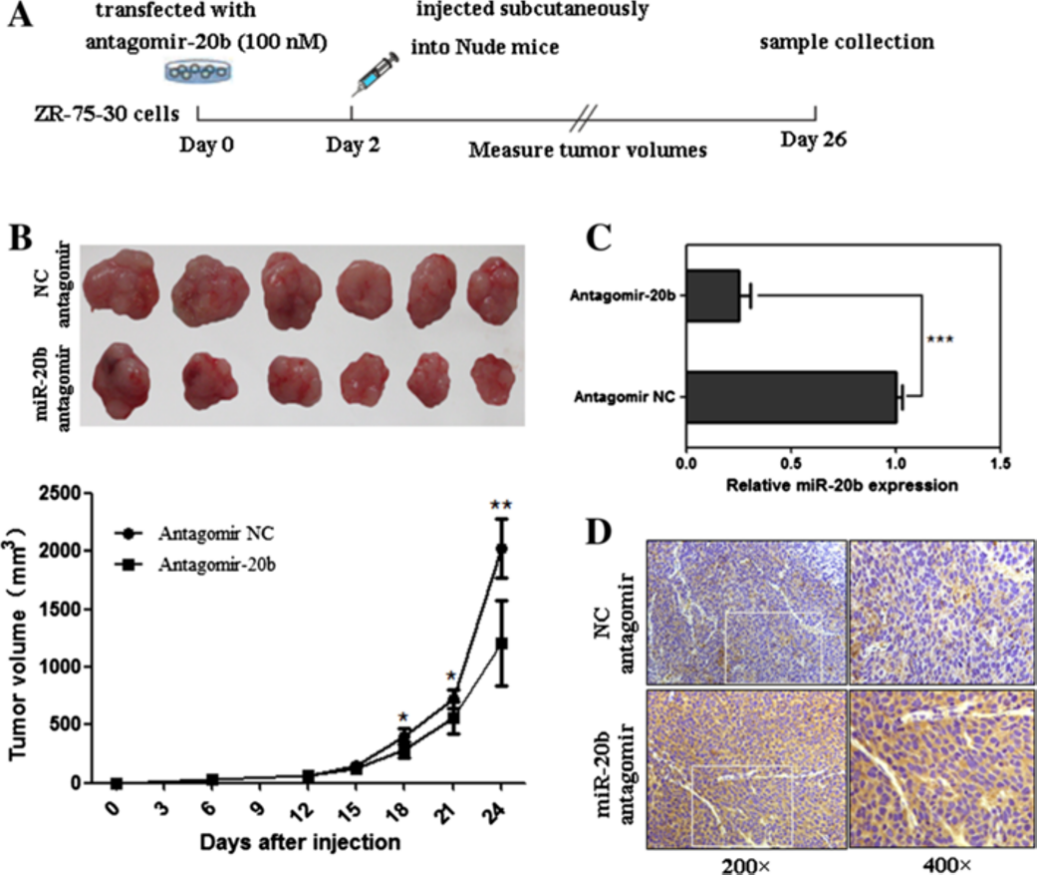
**Knockdown of miR-20b inhibits tumorigenicity of breast cancer cells** ***in vivo*** **. (A)** Schematic diagram of the experimental process. **(B)** Effects of antagomir-20b on tumor formation in nude mouse xenograft model. Female BALB/c nude mice were inoculated with ZR-75-30 cells transfected with antagomir-20b or negative control (antagomir NC) on the right flank (n = 6 mice/group). Tumor volume (V) was monitored by measuring the length (L) and width (W) with vernier caliper and calculated with the formula V = (L × W^2^) × 0.5. After 24 days, the mice were sacrificed and the tumor samples were collected (up-panel). The lower panel shows the volume of the tumors. Data are presented as mean ± SD. *, *P* < 0.05; **, *P* < 0.01 when compared with corresponding negative control. **(C)** qRT-PCR analysis of miR-20b expression in tumor tissues. ***, *P* < 0.001. **(D)** PTEN expression was detected by immunohistochemistry.

In the present study, we found that miR-20b was overexpressed in human breast cancer cells. To further investigate whether the downregulation of miR-20b can suppress tumorigenicity of breast cancer cells, ZR-75-30 breast cancer cells transfected with antagomir-20b or negative control (NC) were subcutaneously inoculated into BALB/c nude mice and their tumorignicity was investigated. Compared with the NC-transfected group, cells transfected with antagomir-20b revealed a delayed tumor formation time and a significant reduction in the tumor size, indicating a potential tumor suppressive effect of antagomir-20b *in vivo* (Figure 7B). The results also showed that miR-20b expression level was significantly decreased in tumor tissues of antagomir-20b group (Figure 7C). Inversely, the increased level of PTEN protein expression was detected in tumor tissues of antagomir-20b group by immunohistochemistry as compared with the NC group (Figure 7D). These results indicated that miR-20b knockdown could suppress the breast tumor growth *in vivo*.

## Discussion

MicroRNAs are known to regulate the expression of genes involved in a wide range of biological processes, such as tumor development, proliferation, apoptosis and stress response [3, 19]. Considering the function of miRNAs, their imbalance expectedly contributes to substantial cell physiological and pathological processes and is ultimately participated in tumorigenesis and the tumor progression of multiple human cancers. In this study, we observed that upregulation of miR-20b in human breast cancer was associated with PTEN protein expression level. MiR-20b could downregulate PTEN protein expression by directly targeting the 3′-UTR of PTEN. Upregulation of miR-20b significantly promoted ZR-75-30 and MCF-7 breast cancer cells viability, colony formation, and led to enhancing DNA synthesis. Conversely, downregulation of miR-20b could significantly inhibit these processes in ZR-75-30 and MCF-7 cells. In addition, knockdown of miR-20b expression could suppress the cell cycle progression in MCF-7 breast cells. Moreover, miR-20b knockdown could suppress tumorigenicity of breast cancer cells *in vivo*. These results suggest that miR-20b functions as an oncomir by contributing to breast tumorigenesis partly via regulating PTEN expression at the posttranscriptional level.

Although miR-20b was reported to involve in the pathogenesis of breast cancer, the molecular mechanism has not been clearly elucidated. A study by *Li et al.* indicated that one of the most important facets underlying metastatic heterogeneity of breast cancer is the differential distribution of miR-20a and miR-20b and their regulation of target proteins, such as VEGF-A and HIF-1α [6]. *Cascio et al.* reported that miR-20b could regulate the expression of vascular endothelial growth factor (VEGF) in MCF-7 breast cancer cells under normoxic and hypoxia-mimicking conditions, which was mediated by hypoxia inducible factor 1 (HIF-1) and signal transducer and activator of transcription 3 (STAT3) [13]. *Li et al.* reported that ionizing radiation, a known breast carcinogen, could trigger the differential expression of miR-20b in mammary tissues. The zinc finger transcription factor early growth response-1 (EGR1) is a crucial player in the transcriptional control of miR-20b, and miR-20b may in turn function as an oncogene by contributing to breast tumorigenesis [14]. In this study, we further investigated the function and possible mechanisms of miR-20b in regulating some biological properties of breast cancer cells. The results obtained from gain-of-function and loss-of-function approaches showed that miR-20b could enhance the cell viability, colony formation and DNA synthesis of breast cancer cells *in vitro*. Moreover, in the study of nude mice model, we validated that knockdown of miR-20b expression level could suppress the tumorigenesis of breast cancer cells *in vivo*. These findings suggested that deregulation of miR-20b played an important role in promoting carcinogenesis and progression of breast cancer.

To explore the molecular mechanism of miR-20b upon the growth of breast cancer cells, we confirmed that miR-20b could directly bind to the 3′-UTR of PTEN and suppress the translation. PTEN is originally identified as a multifunctional tumor suppressor frequently loss in various human cancers [15]. It functions as a negative regulator of the PI3K/Akt pathway via dephosphorylation of phosphatidylinositol‑3,4,5‑trisphosphate (PIP3), ultimately participating in regulation of the cell cycle, proliferation, apoptosis, migration, invasion and metastasis during cancer progression [20]. Recently, PTEN has been shown to be regulated by miRNAs in multiple cancers, including glioma [21], colorectal carcinoma [22], hepatocellular carcinoma [23] and ovarian cancer [24]. Especially, *Wu et al.* reported that miR-32 could enhance the growth, migration, and invasion in colorectal carcinoma cells by regulating PTEN expression [22]. *Wang et al.* found that miR-21 modulated chemosensitivity of breast cancer cells to doxorubicin by targeting PTEN [25]. Liang and his team found that miR-19 involved in multidrug resistance through modulation of PTEN in breast tumor cells [26]. In addition, *Ma et al.* demonstrated that overexpressed microRNA-301a in breast cancer promoted tumor metastasis by targeting PTEN and activating Wnt/beta-catenin signaling [27]. *Li et al.* reported that miR-20b downregulated PTEN expression in HCC1806 cells [14]. A newly study also showed that miR-20b, −21, and -130b were involved in suppression of PTEN expression in colorectal cancer [28]. In this study we also determined that miR-20b inhibited PTEN protein expression by directly binding to the 3′-UTR of PTEN in ZR-75-30 and MCF-7 breast cancer cells. Interestingly, overexpression of miR-20b in ZR-75-30 and MCF-7 breast cancer cells did not significantly change the mRNA expression of PTEN, indicating that miR-20b regulates PTEN expression at the posttranscriptional level. Above all, these studies showed that miR-20b served as an important oncomir in promoting multiple cancer cells growth through regulating tumor suppressor PTEN expression.

## Conclusions

The present study uncovered an oncogenic function of miR-20b that was frequently overexpressed in breast cancer tissues and cell lines. MiR-20b serves as an oncomir that plays an important role in the growth of breast cancer cells partly by targeting tumor suppressor PTEN at the posttranscriptional level. Notably, knockdown of miR-20b expression displays anti-tumor effects both *in vitro* and *in vivo*. Therefore, miR-20b may be a novel diagnostic marker and potential therapeutic target in breast cancer.

## Materials and methods

### Breast cancer tissue specimens

Human breast cancer specimens and matching normal breast tissue samples were surgically resected from 23 patients who had undergone surgery in the breast surgery department at the First Affiliated Hospital of Xiamen University (from 2012 to 2013). The average age at time of diagnosis was 54 years (range: 43–72 years). None of the patients had received preoperative treatment, such as radiotherapy or chemotherapy.

This study was approved by the Ethics Committee of the First Affiliated Hospital of Xiamen University, Xiamen, Fujian, China. All patients provided written informed consent.

### Cell culture and transfection

Human normal mammary epithelial cell line MCF-10A was cultured in DME/F12 medium (Gibco, USA) containing 5% horse serum, 10 μ g/ml insulin, 20 ng/ml EGF, 100 ng/ml choleratoxin, 0.5 μg/ml hydrocortisone, 100 IU/ml penicillin and 100 μg/ml streptomycin. Human breast cancer cell lines MCF-7 and T-47D were maintained in DMEM medium (Gibco) supplemented with 10% fetal bovine serum (FBS; Gibco), 100 IU/ml penicillin and 100 μg/ml streptomycin. Other human breast cancer cell lines ZR-75-30 and SK-BR-3 were cultured in RPMI-1640 medium (Gibco) supplemented with 10% FBS, 100 IU/ml penicillin and 100 μg/ml streptomycin in humidified 5% CO_2_ at 37°C. MiR-20b mimics, miR-20b inhibitor, antagomir-20b and their negative control were synthesized by Ribobio (RiboBio Co. Ltd., Guangzhou, China) [29].

The miR-20b gain-of-function study was performed using miR-20b mimics (50 nM) and its negative control (50 nM) on the breast cancer cell lines. The loss-of-function study was performed with miR-20b inhibitor (100 nM) and its negative control (100 nM) on the cell lines. The cells were transfected using Lipofectamine RNAiMAX reagent (Invitrogen, USA) in accordance with the manufacturer’s instructions.

### Quantitative real-time RT-PCR (qRT-PCR)

Total RNA was extracted using TRIzol reagent (Invitrogen) according to the manufacturer’s instructions. For mature miR-20b expression analysis, Bulge-loop™ miRNA qRT-PCR Primer Sets (one RT primer and a pair of qPCR primers for each set) specific for miR-20b were designed by Ribobio (Guangzhou, China). MiRNA bulge-loop was reverse transcribed using the PrimeScript RT reagent Kit (Takara, Dalian, China) and quantified by qPCR using the SYBR Premix Ex Taq (TaKaRa) as described previously [30]. U6 was used as the internal control. mRNA quantification was similar as miRNA quantification, except that cDNA was reverse transcribed using oligo(dT)_18_ primer. All quantitative real-time PCR was performed on the Light Cycler 480 system (Roche, USA). The primers used were as the following: PTEN-S: 5′-TAGAGGAGCCGTCAAATC-3′; PTEN-AS: 5′-ATCAGAGTCAGTGGTGTC-3′; GAPDH-S: 5′-GGAAGGTGAAGGTCGGAGTCA-3′; GAPDH-AS: 5′-GTCATTGATGGCAACAATATCCACT-3′. The specific gene expression was calculated by using the comparative 2^-△△CT^ method with GAPDH as calibrator [31].

### Western blot analysis

Total protein from breast cancer tissues, adjacent normal tissues, and cultured cells were extracted using RIPA buffer (Thermo, USA). Whole-cell lysates (30 ~ 40 μg) were separated by 10% sodium dodecyl sulfate polyacrylamide gel electrophoresis (SDS-PAGE), and the proteins were transferred to polyvinylidene difluoride membranes by electroblotting. Non-specific binding was blocked by incubating the membranes in 5% non-fat milk in TBST (50 mmol/L Tris–HCl, 150 mmol/L NaCl, 0.1% Tween-20) for 1 h at room temperature. Membranes were incubated overnight at 4°C with PTEN antibody (Cell Signaling Technology, USA) and β-actin antibody (Santa Cruz, USA) both at a 1:1000 dilution. The membranes were subsequently incubated with a horseradish peroxidase-conjugated secondary antibody (1:10000; Pierce, Rockford, IL, USA) for 1 hour at room temperature and visualized using enhanced chemiluminescence (Pierce) and X-ray film.

### Luciferase reporter assays

The putative binding sites of miR-20b in the 3′-UTR of the human PTEN gene were predicted by combinatorial utilization of three different algorithms, including TargetScan (http://www.targetscan.org/), miRanda (http://www.microrna.org/), and PicTar (http://pictar.mdc-berlin.de/). Direct targeting of the PTEN 3′-UTR was determined by cloning of the 3′-UTR seed regions and mutated seed regions into separate pGL3 Luciferase Reporter vectors (Promega). The primers selected were as the following: PTEN-wt-S: 5′-CTAGAAATTAGGATTAATAAAGATGGCACTTTCCCGTTTTATTCCAGTT-3′; PTEN-wt-AS: 5′-TTTAATCCTAATTATTTCTACCGTGAAAGGGCAA AATAAGGTCAAGATC-3′; PTEN-mut-S: 5′-CTAGAAATTAGGATTAATAAAGATGTTTGCACCCCGTTTTATTCCAGTT-3′; PTEN-mut-AS: 5′-TTTAATCCTAATTATTTCTACAAACGTGGGGCAAAATAAGGTCAAGATC-3′. The reporter vector plasmid was transfected into breast cancer cells using Lipofectamine 2000 (Invitrogen) according to the manufacturer’s instructions. To correct for the transfection efficiency, a luciferase reporter vector (pRL-TK Vector, Promega) without miR-20b target was transfected in parallel. Renilla and firefly luciferase activities were measured using the Dual Luciferase Reporter Assay System (Promega) and luminescence recorded on a Synergy Multi-Mode Plate Reader (Boitek, USA). MiR-20b function was expressed as a percent reduction in the luciferase activity of cells transfected with the reporter vector containing the miR-20b target sequences compared with cells transfected with the vector without the miR-20b target.

### Cell viability assays and colony formation assays

For the cell viability assay, 4 × 10^3^ cells per well were seeded into a 96-well plate in quintuplicate, the cell growth was measured by CellTiter 96® AQueous One Solution Cell Proliferation Assay (MTS; Promega, USA) after the indicated periods. Absorbance was measured at 490 nm using a microplate reader. For colony formation assays, cells were plated on 6-well (0.5 × 10^3^ cells per plate) and cultured for 7 days. The colonies were stained with 1.0% crystal violet solution for 30 min. Colonies > 50 μ m in diameter were counted.

### 5-Ethynyl-2′-deoxyuridine (EdU) labeling assays

Cells were transfected with miR-20b mimics or miR-20b inhibitor in 96-well plates. Forty-eight hours after transfection, 5-Ethynyl-2′-deoxyuridine (EdU) (50 μM) (Cell-LightTM EdU Apollo567 In Vitro Kit, RiboBio) was added and the cells were cultured for an additional 2 h. The cells were then fixed in 4% paraformaldehyde at room temperature for 30 min. After permeabilization with 0.5% Triton-X, the cells were incubated with 1 × Apollo reaction mixture for 30 min. Subsequently, the cells were stained with Hoechst 33342 for 30 min at room temperature. Images were taken and analyzed using Nikon Eclipse 50i fluorescent microscope (Tokyo, Japan). EdU positive cells were calculated with (EdU add-in cells/Hoechst stained cells) × 100%.

### Cell cycle analysis

To analyze the cell cycle distribution, DNA content was determined by flow cytometry using propidium iodide (PI) staining. Briefly, cells were harvested, washed twice with PBS and fixed in 75% ethanol (in 0.01 mol/L PBS) at 4°C for at least 12 h. After being centrifuged, the cells were incubated in PBS containing 100 μg/mL DNase-free RNase A at 37°C for 30 min and subsequently stained with 50 μg/mL PI on ice for 30 min in the dark. The cells were analyzed by flow cytometry with a Coulter EPICS XLTM flow cytometer from Beckman Coulter Inc. (Brea, CA).

### *In vivo*tumorigenicity assays

Healthy female BALB/c Nude mice (4 weeks old) were obtained from the Xiamen University Laboratory Animal Centre in Xiamen, China. Animals were maintained for 7 days in a conventional animal care unit before the start of the study. Animal experiments were performed according to ethical guidelines of animal experimentation. ZR-75-30 cells transfected with antagomir-20b or negative control (3 × 10^6^ in 0.2 ml PBS) were injected subcutaneously into the right flank of each mouse (6 mice/each group). Tumor volume (V) was monitored by measuring the length (L) and width (W) with vernier caliper and calculated with the formula V = (L × W^2^) × 0.5. After 24 days, the mice were sacrificed and the tumor samples were processed for qRT-PCR and immunohistochemistry examination.

### Statistical analysis

Data were expressed as the mean ± standard deviation (SD), and statistical analysis was performed using SPSS software (version 16.0, Chicago, USA). After testing the data for normal distribution and equal variance, differences between two groups were analyzed by the unpaired Student’s t-test, and differences between multiple groups were analyzed by ANOVA. *P* < 0.05 was considered statistically significant.

## Electronic supplementary material

Additional file 1: Figure S1: PTEN protein expression is negatively correlated with miR-20b expression. Statistical and correlation analyses of PTEN protein level and miR-20b expression in ten freshly prepared normal human breast tissues and ten human breast cancer tissues. (TIFF 218 KB)

Additional file 2: Table S1: Effect of miR-20b on cell cycle in MCF-7 cells. (PDF 43 KB)
