# Supplementary figures and images for: MicroRNA-20b promotes cell growth of breast cancer cells partly via targeting phosphatase and tensin homologue (PTEN)
Source: Cell Biosci. 2014 Oct 14;4:62. doi: 10.1186/2045-3701-4-62 (PMC4216355; doi:10.1186/2045-3701-4-62)

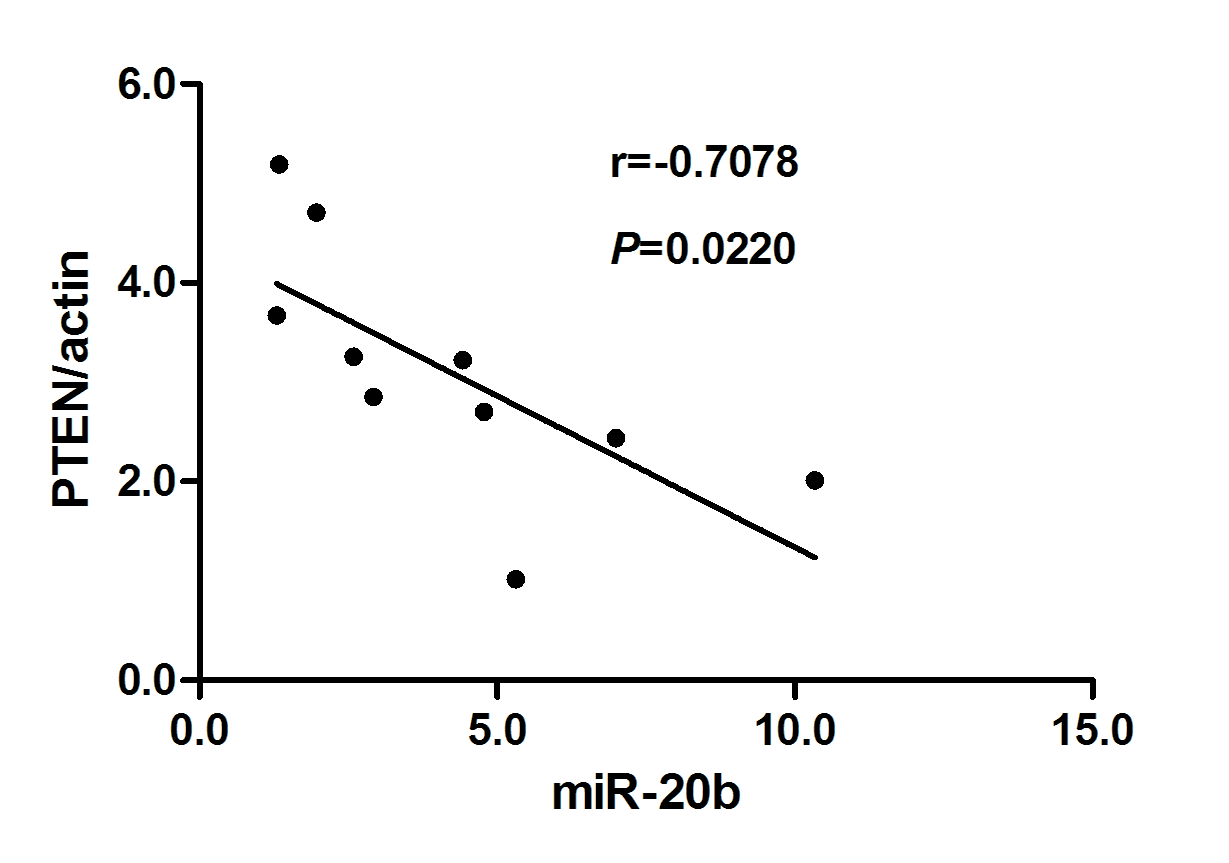

Supplement: Supplementary file 1 — Additional file 1: Figure S1: PTEN protein expression is negatively correlated with miR-20b expression. Statistical and correlation analyses of PTEN protein level and miR-20b expression in ten freshly prepared normal human breast tissues and ten human breast cancer tissues. (TIFF 218 KB) [file 13578_2014_189_MOESM1_ESM.tiff]
