# Supplementary material for: MicroRNA-20b promotes cell growth of breast cancer cells partly via targeting phosphatase and tensin homologue (PTEN)
Source: Cell Biosci. 2014 Oct 14;4:62. doi: 10.1186/2045-3701-4-62 (PMC4216355; doi:10.1186/2045-3701-4-62)
Supplement: Supplementary file 2 — Additional file 2: Table S1: Effect of miR-20b on cell cycle in MCF-7 cells. (PDF 43 KB) [file 13578_2014_189_MOESM2_ESM.pdf]

Tabe S1. Effect of miR-20b on cell cycle in MCF-7 cells.

Group	G_0/G_1 (%)	S (%)	G_2/M (%)	Proliferation index
Negative control	43.83±5.72	38.60±6.80	17.57±3.16	0.56±0.06
miR-20b inhibitor	62.81±2.38	28.54±2.06	8.65±0.59	0.37±0.02[a]

Proliferation index= (S phase + G_2/M phase)/ (G_0/G_1 phase + S phase + G_2/M phase);

[a] $P < 0.01$, when compared with negative control.
